# Overexpression of Soybean-Derived Lunasin in Wheat and Assessment of Its Anti-Proliferative Activity in Colorectal Cancer HT-29 Cells

**DOI:** 10.3390/ijms21249594

**Published:** 2020-12-16

**Authors:** Xin Fan, Peiyou Qin, Yuqiong Hao, Huimin Guo, Christophe Blecker, Nadia Everaert, Guixing Ren

**Affiliations:** 1 Institute of Crop Science, Chinese Academy of Agricultural Sciences, No. 80 South Xueyuan Road, Haidian, Beijing 100081, China; fx_926@163.com (X.F.); qinpeiyou@caas.cn (P.Q.); haoyuqiong334@163.com (Y.H.); guohuimin0208@sina.com (H.G.); 2 Gembloux Agro-Bio Tech, University of Liège, 5030 Gembloux, Belgium; christophe.blecker@uliege.be (C.B.); nadia.everaert@uliege.be (N.E.)

**Keywords:** transformation, transgenic, UPLC-MS/MS, cytotoxic, apoptosis

## Abstract

Lunasin is a soybean-derived peptide that exhibits anticancer bioactivity in different cancer cells and has been identified in different plants. However, recent studies revealed through molecular and chemical analyses that lunasin was absent in wheat and other cereals. In this study, the soybean-derived lunasin was cloned into pCAMBIA3300 and we transferred the expression vector into wheat via an *Agrobacterium*-mediated transformation. The identification of transgenic wheat was detected by polymerase chain reaction, Western blot analysis, and ultra-performance liquid chromatography with tandem mass spectrometry. An enzyme-linked immunosorbent assay showed that lunasin content in transgenic wheat L32-3, L32-6, and L33-1 was 308.63, 436.78, and 349.07 µg/g, respectively, while lunasin was not detected in wild-type wheat. Lunasin enrichment from transgenic wheat displayed an increased anti-proliferative activity compared with peptide enrichment from wild-type wheat in HT-29 cells. Moreover, the results of a real-time quantitative polymerase chain reaction showed a significant elevation in p21, Bax, and caspase-3 expression, while Bcl-2 was significantly downregulated. In conclusion, soybean-derived lunasin was successfully expressed in wheat via *Agrobacterium*-mediated transformation and may exert anti-proliferative activity by regulating the apoptosis pathway in HT-29 cells, which provides an effective approach to compensate for the absence of lunasin in wheat.

## 1. Introduction

The beneficial effects of whole grains have attracted widespread attention. Wheat is one of the most important crops globally and provides vegetable proteins for humans. Previous studies have found that a chemopreventive peptide, lunasin, possesses antioxidant, anti-inflammatory, and anticancer activities in wheat [1,2,3,4]. Lunasin is a 44-amino acid soybean derived peptide that contains nine Asp-residues at the carboxyl end, an Arg-Gly-Asp (RGD) cell adhesion motif, and a conserved helix region [5]. Similar to the lunasin isolated from the soybean, lunasin extracted from wheat also exhibits the core histone H3- and H-acetylation inhibitory properties that lead to cell death, which occurs to cells undergoing transformation [1,6].

The anti-cancer bioactivity of lunasin has been demonstrated in previous studies. Galvez et al. revealed that synthetic lunasin is capable of inhibiting chemical carcinogen-induced transformation of murine fibroblast cells to cancerous foci [4]. Lunasin also suppresses foci formation in NIH/3T3 cells by increasing the p21 expression level [7]. Other studies have focused on the effect of lunasin in colon cancer. Dia et al. found that lunasin from soybean has the potential to promote apoptosis in HT-29 colon cancer cells [8]. Likewise, lunasin served an anti-cancer function in other human colon cancer cells, influencing the expression associated with extracellular matrix and cell adhesion genes [9]. Recently, evidence suggested that lunasin exerts its chemopreventive activity on colorectal cancer by regulating the parental and tumorsphere-derived subsets in HCT-116 cells [10]. Although lunasin has been found in different crops and possesses anti-cancer bioactivity, the DNA sequence and transcript of lunasin from wheat and other cereals differed from that in soybean. In addition, the results of molecular and chemical analyses showed that there were no lunasin-related sequences in analyzed wheat samples [11].

With the rapid advances in genome editing technology, this provides a new approach to improve crop varieties in the field. Until recently, different types of genes have been transferred into the wheat genome, and most of them were related to stress resistance and physicochemical characterization. The overexpression of *Arabidopsis* OPR3, AtWRKY30 transcription factor, and wheat aquaporin gene *TdPIP2;1* effectively enhanced wheat tolerance to freezing, heat stress, and drought, respectively [12,13,14]. Modification of high-molecular-weight glutenin subunit 1Ax1, low-molecular-weight glutenin subunit LMW-N13, and 1Dy12 subunit improved the quality of wheat [15,16,17]. Furthermore, a synthetic *avidin* gene was introduced into spring wheat (*Triticum aestivum* L.) to improve its resistance to the stored product insect [18]. Zhang et al. overexpressed a rice gene *OsHMA3* in wheat, which led to a significant reduction in cadmium accumulation [19]. Nevertheless, few studies have attempted to enhance the content of functional proteins and peptides in wheat.

Due to the low expression or absence of lunasin in different cereals, soybean-derived lunasin has been introduced into *Escherichia coli*, *Pichia pastoris*, rice, and soybean [20,21,22,23,24]. However, lunasin has not been transferred into wheat yet, partially because of its much lower transformation efficiency [25]. In this work, lunasin from soybean was successfully transferred into the wheat genome via the modified *Agrobacterium*-mediated method to generate transgenic wheat. The identification of lunasin in transgenic wheat was conducted by polymerase chain reaction (PCR), Western blot analysis, and ultra-performance liquid chromatography with tandem mass spectrometry (UPLC-MS/MS). Enzyme-linked immunosorbent assays (ELISA) were used to detect the content of lunasin. The bioactivity of lunasin on colorectal cancer was evaluated in human CRC HT-29 cells.

## 2. Results

### 2.1. Molecular Analysis of Transgenic Wheat

**Figure 1:**
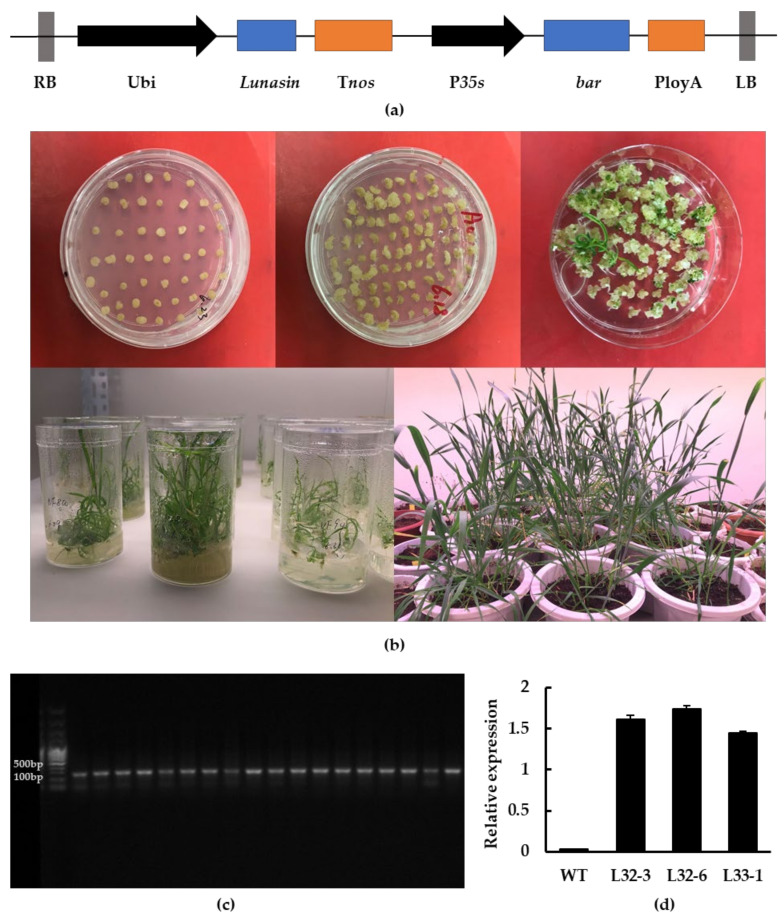
Generation of transgenic wheat and molecular identification. (**a**) Construction of the lunasin expression vector; lunasin was driven by the Ubi promoter. (**b**) *Agrobacterium*-mediated transformation of wheat immature embryos. (**c**) PCR identification of transgenic lines. (**d**) Lunasin expression analysis in transgenic lines L32-3, L32-6, L33-1.

Through *Agrobacterium*-mediated transformation, transgenic wheat calluses expressing the lunasin gene under the control of maize ubiquitin (Ubi) promoter were obtained with glyphosate-resistance screening. Wheat seedlings were obtained after one month and the wheat seeds were harvested six months later, as described in the Methods section (Figure 1a,b). The result of PCR identification showed that the fragment produced by transgenic wheat was around 120 bp, while it was not detected in wild-type wheat (Figure 1c). Transgenic wheat lines L32-3, L32-6, and L33-1 were chosen for further experiments based on their lunasin expression level and seed quantity. As shown in Figure 1d, lunasin expression in L32-3, L32-6, and L33-1 was 1.611 ± 0.046, 1.737 ± 0.044, and 1.447 ± 0.021, respectively, and there was no detection of lunasin in wild-type wheat.

### 2.2. Identification and Content of Lunasin in Transgenic Wheat

**Figure 2:**
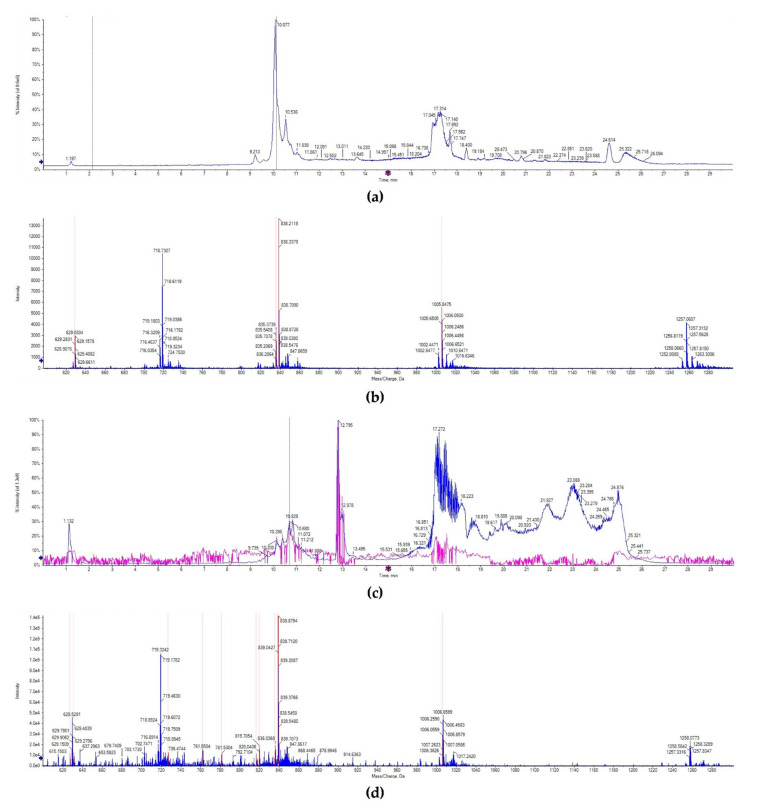
UPLC-MS/MS analysis. (**a**) The chromatogram of the lunasin standard. (**b**) Mass spectrum acquired from the peak at 10.138 min in the chromatogram of the lunasin standard, [M + 7H]^7+^ at 719.32 m/z, [M + 6H]^6+^ at 838.70 m/z, [M + 5H]^5+^ at 1006.65, and [M + 4H]^4+^ at 1257.31 m/z. (**c**) The chromatogram of lunasin extracts from transgenic wheat. (**d**) Mass spectrum acquired from the peak at 10.681 min in the chromatogram of lunasin extracts from transgenic wheat, [M + 7H]^7+^ at 719.32 m/z, [M + 6H]^6+^ at 838.71 m/z, [M + 5H]^5+^ at 1006.65, [M + 4H]^4+^ at 1258.07 m/z.

The lunasin identification in wheat was detected by UPLC-MS/MS. Consistent with previous studies, the chromatogram of lunasin standard showed that lunasin ions contain a multicharged form ([M + 7H]^7+^ at 719.32 m/z, [M + 6H]^6+^ at 838.70 m/z, [M + 5H]^5+^ at 1006.65, and [M + 4H]^4+^ at 1257.31 m/z), and exhibited a peak at the retention time of 10.138 min (Figure 2a,b). However, the chromatogram signal of the lunasin-enriched fraction of transgenic wheat (LETW) was slightly complex. At the peak with the retention time of 10.681 min, the mass spectrum showed a similar multicharged profile (Figure 2c,d). Moreover, there was no lunasin ions deceted in wild-type wheat (Supplementary Figure S1). These results proved that the lunasin expressed in transgenic wheat was the same as the synthetic lunasin.

**Figure 3:**
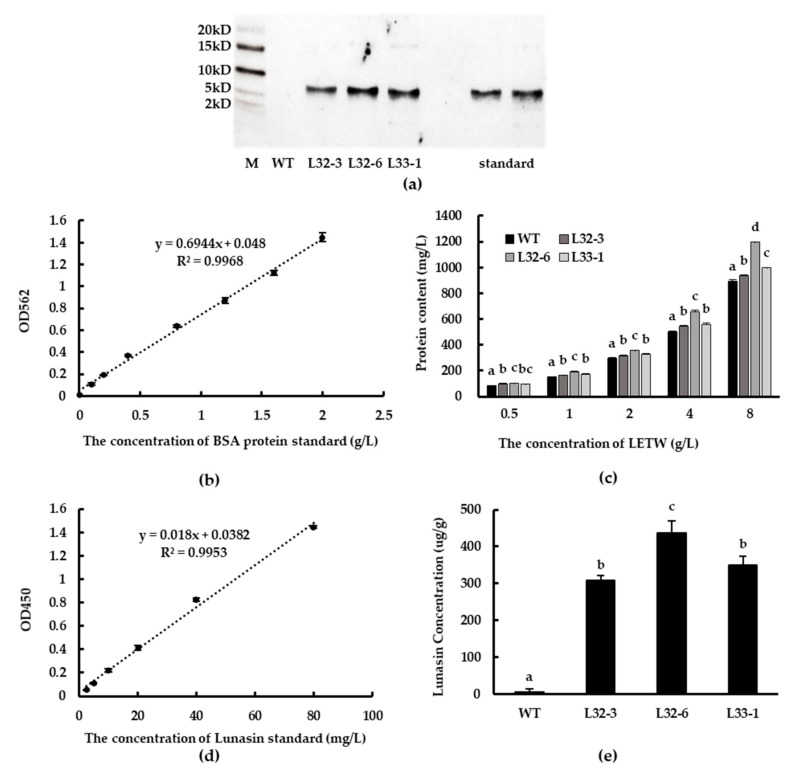
Western blot analysis and detection of lunasin content. (**a**) Lunasin of transgenic wheat detected by western blot. (**b**) BSA standard curve for the BCA protein assay. (**c**) The protein content of lunasin-enriched fraction of transgenic wheat (LETW) and peptides-enriched fraction of wild-type wheat (PEWW) at different concentrations. (**d**) Lunasin standard curve detected by ELISA. (**e**) Lunasin content in wild-type wheat and different transgenic lines. Data are shown as the means of three independent experiments; different letters above the columns between different lunasin-overexpression lines show significant differences (*p* < 0.05).

Western blot analysis showed that lunasin was successfully expressed at the translation level in transgenic lines L32-3, L32-6, and L33-1, while synthetic lunasin standard was also detected as a control (Figure 3a). The protein content of LETW was tested using BCA Protein Assay Kit (GenStar BioSolutions Corporation, Beijing, China). The standard curve of BSA protein (0–2 g/L) is shown in Figure 3b. There was a significant increase in protein content of LETW compared to the peptides-enriched fraction of wild-type wheat (PEWW), and the increasing trend was positively correlated with the concentration of LETW (Figure 3c). Using ELISA to engage at least one antibody with an exclusive counterpart antigen is an approach used to improve sensitivity and specificity [26]. Subsequently, the lunasin content of LETW was measured via a modified ELISA method. The standard curve of lunasin was linear and the relationship between absorbance and lunasin content was calculated based on the range 2.5–80 mg/L. The linear equation was *y* = 0.018*x* + 0.0447, *R*^2^ = 0.9931 (Figure 3d). The lunasin content of LETW in L32-3, L32-6, and L33-1 was 308.63, 436.78, and 349.07 µg/g, respectively. There was no lunasin detected in wild-type wheat (Figure 3e).

### 2.3. Effects of LETW on Cell Proliferation in HT-29

**Figure 4:**
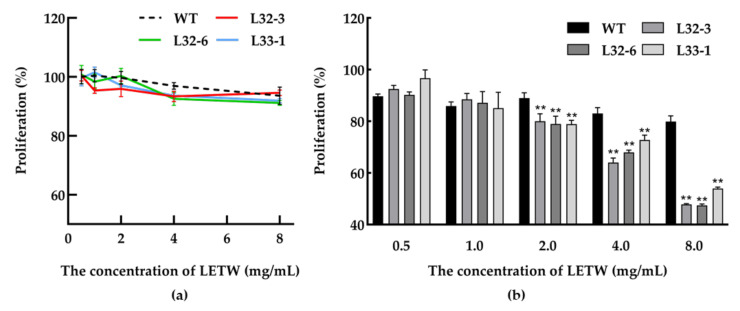
Cytotoxic effect and anti-proliferative activity of LETW on colorectal cancer cells. (**a**) Dose-dependent effect of LETW on HT-29 cells after 24 h treatment; (**b**) the cell proliferation of HT-29 cells was significantly inhibited by LETW after 72 h incubation. ** *p* < 0.01 indicate significant and highly significant differences between the LETW and PEWW treatments, respectively.

In this study, the toxicity test of LETW was measured in HT-29 cells. The number of viable cells was calculated by an CCK-8 assay after being incubated with 0.5–8 g/L LETW or PEWW for 24 h. As shown in Figure 4a, neither LETW nor PEWW showed cytotoxic effects with different experimental gradients (0.5–8 g/L) on HT-29 cells. To evaluate the anti-proliferative activity of LETW on colon cancer, the CCK-8 assay was performed in human HT-29 cells. As shown in Figure 4b, after 72 h of incubation, there was no difference in HT-29 cells under 0.5–1 g/L LETW and PEWW treatment. Once the dose of LETW was increased to 2 g/L, the proliferation of HT-29 cells was significantly inhibited. Moreover, the inhibition rate under LETW treatment reached 47.44–53.98% of the PEWW treatment group at a content of 8 g/L.

### 2.4. Expression Analysis of Apoptosis-Related Genes under LETW Treatment in HT-29

**Figure 5:**
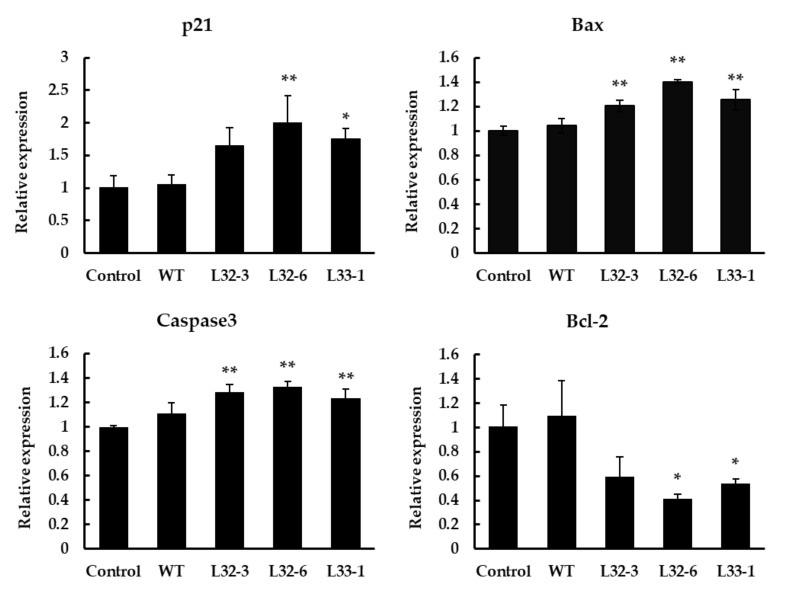
Gene expression analysis using RT-qPCR in HT-29 cells after 48 h of treatment with 4 g/L LETW. Quantitative analysis of gene expression results showed that the expression of p21, Bax, and caspase-3 was significantly increased. However, the gene expression of Bcl-2 was inhibited by LETW. The gene expressions were shown in mean ± SD. * *p* < 0.05 and ** *p* < 0.01 indicate significant and highly significant differences of the test to the control, respectively.

Bcl-2 and Bax have previously been shown to play a key role in mediating the mitochondrial pathway, which leads to the activation of caspase-9, -3, and -7 [27]. After treatment with LETW in HT-29 cells for 48 h, the marker genes in cell apoptotic pathways were implicated. As shown in Figure 5, the results showed a significant elevation in the p21, Bax, and caspase-3 expression after treatment, while the expression of antiapoptotic gene Bcl-2 was significantly downregulated. Compare to the control group, the expression of p21, Bax, and caspase-3 with LETW treatment were upregulated by 1.65–2.01, 1.20–1.40, and 1.24–1.33 fold, respectively. In contrast, the antiapoptotic protein Bcl-2 was inhibited by LETW, and was approximately 0.41–0.59-fold lower than the control group.

## 3. Discussion

Lunasin, a peptide originally identified in soybean, has potential chemopreventive activity to alleviate different types of cancer. In previously published studies, researchers focused on how to extract lunasin from different cereals or plants, a process which was unstable and cumbersome. The transformation has provided solutions for plant improvement and was successfully applied in different organisms. Lunasin was expressed through an *Escherichia coli* T7 expression system with the use of vector pET29a, significantly inhibiting histone acetylation [21]. Kyle et al. used a pET28 vector to express a CBD-lunasin fusion with a hexahistidine tag and tobacco etch virus protease site, and lunasin can be released by TEV protease cleavage [22]. Lunasin has also been introduced into rice and soybean by *Agrobacterium*-mediated transformation; the lunasin content in transgenic rice was 1.01 mg/g, while in transgenic soybean it was in the range 1.32–1.97 mg/g [24,25]. However, it is not easy to express an exogenous gene into hexaploid wheat due to the lower transformation efficiency. Hence, in order to address this limitation, we constructed the expression vector without unnecessary vector backbone DNA in early generations, according to the research of Wang et al. [28], and we decreased the bar gene integration by changing the sizes of the two independent T-DNA regions in the vector [26]. Then, the transgenic wheat lines were generated by the *Agrobacterium*-mediated co-transformation method. The results of PCR and qPCR analysis showed that lunasin was expressed as expected. However, in this study, the lunasin content in transgenic wheat (308.63–436.78 µg/g) was lower than that found in transgenic soybean and transgenic rice. This was likely due to the complex hexaploid wheat genome and the wheat cultivars used. Thus, more efficient gene editing methods and other wheat cultivars suitable for exogenous gene expression should be investigated in future studies.

Lunasin was first detected in wheat by Jeong et al. using liquid chromatography electrospray ionization mass spectrometry (LC–ESI-MS). They demonstrated that lunasin isolated from wheat inhibits core histone acetylation [1]. However, recent studies have cast doubt on the presence of lunasin in wheat. Dinelly et al. reported that lunasin was absent in the studied wheat varieties measured via LC-MS and PCR [12]. Similarly, Mitchell et al. did not identify the same sequence as was detected to encode lunasin in soybean through proteomic and transcriptomic analysis in wheat and the deep alignment in different DNA sequence databases [29]. In this work, immunology experiment results showed that lunasin was indeed absent in wild-type wheat, but active lunasin was detected in transgenic wheat. This finding is consistent with the result of Alaswad et al., who carried out immunological analysis in cereals and other plant species [30]. There may be various factors (cultivars, location, soil conditions, etc.) involved in this disparity of findings. Based on our study, it was feasible to overexpress the soybean lunasin in wheat by genetic transformation. More research is needed to focus on how to optimize the expression system and cultivation conditions of transgenic wheat.

The bioactivity of lunasin has been investigated for many years: Dia et al. reported that lunasin purified from defatted soybean flour displayed no cellular toxicity to CCD33Co normal colon fibroblast up to 100 µM [10]. Moreover, the LET of different concentrations (0.25–4.0 g/L) from lunasin-overexpression soybean did not show toxicity on MDA-MB-231 cells in the research of Hao et al. [25]. However, Dia et al. proved that treatment with a higher dose of lunasin induced cytotoxicity in different colon cancer cell lines, including HCT-116, HT-29, KM12L4, and RKO cells [10]. This finding was recently supported by Fernandez-Tome et al., who revealed that 10 µM lunasin had a significant cytotoxic effect on the cellular growth of HCT-116, and suggested that the different inhibitory potency between synthetic lunasin and plant-purified lunasin might be due to the differences in the secondary and tertiary structures [11]. Although lunasin from soybean and synthetic lunasin was not exactly the same, both exhibit anti-proliferative activity against colon cancer cells [8,10,11]. Those findings are largely consistent with findings of this study. Moreover, the lunasin extracted from transgenic soybean possessed anti-proliferative activity in MDA-MB-231 human breast cancer cells [25]. Liu et al. and Zhu et al. efficiently overexpressed lunasin in *Escherichia coli* and *Pichia pastoris*, respectively [21,23]. Therefore, we proposed a new strategy to produce lunasin in wheat, and the soybean-derived lunasin overexpressed in wheat was able to exert anti-colon cancer activity. Despite considerable evidence showing that lunasin inhibits the growth of cancer cells by participating in cell cycle progression and chromatin modification, the regulation mechanism in these processes still needs further exploration.

Apoptosis is a biological phenomenon controlled by genes highly conserved in evolution to maintain normal growth, development, and tissue homeostasis [31]. According to the earlier studies of Dia et al., lunasin could cause cell arrest at G2/M phase and the mitochondrial-mediated apoptosis in HT-29 cells. Moreover, they also revealed that lunasin modulates the Bax: Bcl-2 ratio by upregulating the anti-apoptotic protein Bax and downregulating the pro-apoptotic protein Bcl-2, which led to the release of cytochrome c, which participates in the apoptotic mitochondrial pathway [8]. In L12120 leukemia cells, lunasin has the ability to arrest the cell cycle at the G2/M phase and induce apoptosis. Moreover, there is a positive correlation between the apoptotic cell number and the expression of caspase-3, -8, and -9 [32].

Although the effect of lunasin on apoptosis has been demonstrated in different cell lines, the anti-cancer bioactivity of lunasin extract from wheat has not been proven because of the evidence of Dinelli et al. suggesting the absence of lunasin in wheat [12]. In this work, the molecular and cell assays have shown the presence of lunasin in transgenic wheat, and confirmed its anti-proliferative activity. Our results are supported by recent studies that it is feasible to overexpress lunasin in different expression systems, including studies on *Escherichia coli*, *Pichia pastoris*, rice, and soybean [21,23,24,25]. We further investigated the effect of LETW on the expression of apoptosis-related genes in colon cancer cells, and the results showed that LETW may inhibit the growth of HT-29 cells by regulating the apoptosis pathway. However, more research is needed to increase the expression level of lunasin in transgenic wheat and to verify its anti-cancer activity using in vivo experiments, which would be meaningful for the development of anti-cancer functional foods.

## 4. Materials and Methods

### 4.1. Regents

Primers were synthesized by BGI Tech Solutions (Beijing Liuhe) Co., Ltd. (Beijing, China) and are shown in Table 1. Direct-load 100 bp DNA ladder and 2 × Taq PCR MasterMix were obtained from GenStar BioSolutions Corporation (Beijing, China). The lunasin standard and rabbit polyclonal primary antibody against the lunasin epitope (EKHIMEKIQGRGDDDDD) were synthesized by Sangon Biotech Corporation (Shanghai, China). Goat anti-rabbit IgG-HRP was purchased from Biodee Biotechnology Co., Ltd. (Beijing, China). Roswell Park Memorial Institute 1640 (RPMI1640), and fetal bovine serum (FBS) were purchased from Sigma-Aldrich (St. Louis, MO, USA). Penicillin and streptomycin were purchased from Mediatech, Inc. (Manassas, VA, USA).

### 4.2. Construction of the Expression Vector

Total RNA was extracted from soybean using a RNAprep Plant Kit (Tiangen Biotech, Beijing, China) and reverse transcribed into cDNA using cDNA Synthesis SuperMix kit (TransGen Biotech, Beijing, China) following the recommended protocol. cDNA was used to amplify the full-length lunasin cDNA sequence containing *Bam*HI and *Sac*1 restriction sites. Forward primer Lunasin-F and Reverse Primer Lunasin-R were used for PCR amplification. The program used for PCR was as follows: 95 °C for 10 min (1 cycle); 95 °C for 30 s, 56 °C for 30 s, 72 °C for 30 s (30 cycles), and 72 °C for 5 min (1 cycle). The PCR products were ligated to vector pCAMBIA3300 (with the *bar* gene), double digested with *Bam*HI and *Sac*1 enzymes, and the lunasin was driven by the Ubi promoter (Figure 1a).

### 4.3. Agrobacterium-Mediated Transformation of Wheat Immature Embryos

Transgenic wheat plants were produced according to the proprietary method described by Ishida et al. All of the media used below were developed according to the protocol of Ishida et al. [33] Immature wheat embryos taken from the sterilized wheat seeds were used as explants for *Agrobacterium*-mediated transformation using *Agrobacterium* strain C58C1 harboring pCAMBIA3300-Lunasin. After 2 days of co-cultivation, embryonic axes were transferred onto WLS-AS medium. After 5 days, the tissues were transferred onto callus induction medium. Two weeks later, callus was cut into halves and cultured in darkness on WLS-P10 medium for 3 weeks. Embryogenic calli were differentiated on LSZ-P5 medium under light and regenerated shoots were transferred in sterilized bottles filled with MSF-P5 medium for root formation (Figure 1b). According to the growth of seedlings, well-developed plants were transplanted into pots and cultivated in a greenhouse.

### 4.4. Screening for Transgenic Wheat

The glyphosate-tolerance of transgenic wheat plants was assayed by herbicide spraying, and PCR detection was performed at the same time. The genomic DNA was extracted by DNAsecure Plant Kit (TianGen Biotechnology Co., Ltd., Beijing, China). The PCR primers were designed according to the *lunasin* and *bar* sequences by Primer3 (v. 0.4.0). The PCR amplification procedure was as follows: 95 °C for 30 s, 56 °C for 30 s, 72 °C for 30 s (30 cycles), and 72 °C for 5 min (1 cycle). The positive transgenic plants were grown to obtain the next generation. T2 positive transgenic wheat lines were also screened by PCR and verified by Sanger sequencing. Total RNA was extracted, and cDNA was obtained as described in Section 4.2. The expression of lunasin in T2 transgenic wheat lines was examined by RT-qPCR using the TransStart Top Green qPCR Supermix kit (TransGen Biotech, Beijing, China) and the 7500 Real-Time PCR System (Applied Biosystems, Foster City, CA, USA). Transgenic wheat was selected based on the expression results and was harvested for seeds for subsequent experiments.

### 4.5. Isolation, Purification, and Identification of Lunasin from Wheat

To isolate lunasin, wheat seeds were ground to flour using a FOSS CT293 Cyclotec (Foss Tecatur AB, Hillerød, Denmark), and 1 g flour was extracted with 10 mL PBS (pH 7.4), added with 1% (*v*/*v*) protease inhibitor cocktail (including aprotinin, bestatin, leupeptin and pepstatin A) (Sigma, St. Louis, MO, USA) by shaking for 48 h at 4 °C. The resulting mixture was centrifuged at 12,000× *g* for 30 min and the supernatants were collected. The purification process was based on the method of Hao et al. [25]. The modified method was as follows: Supernatants were centrifuged at 4000× *g* for 30 min in Vivaspin Turbo 15 RC (Sartorius AG, Göttingen, Germany) and dialyzed for 24 h at 4 °C in Spectra membrane (MWCO: 3000). Finally, LETW and PEWW were freeze-dried for further experimentation.

Lunasin was identified by western blot analysis based on the method of Hao et al. [25]. All freeze-dried samples were dissolved in pure water and separated in 12% *v*/*v* sodium dodecyl sulfate-polyacrylamide gel. Then, we transferred all samples to the nitrocellulose membrane (0.22 µm) using Bio-Rad Trans-blot apparatus (Bio-Rad, Cambridge, MA, USA). After 30 min, the membrane was blocked in blocking buffer (0.05% *v*/*v* Tween 20, 5% *w*/*v* nonfat dry milk) for 1 h. When the blocking was done, the membrane was washed with Tris Buffered saline Tween (TBST) 3 times. The primary rabbit polyclonal antibody and goat anti-rabbit IgG-HRP were used to conduct incubation steps for 1 h and 45 min, respectively. After hybridization, the membrane was incubated in Immobilon ECL Ultra Western HRP Substrate (Millipore Corporation, Burlington, MA, USA) for 20 min. Finally, the detection was conducted through a Tanon 5200 (Tanon Corporation, Shanghai, China).

### 4.6. UPLC-MS/MS Analysis

Lunasin extract from wheat was identified using a SCIEX TripleTOF6600 mass spectrometer connected to a UPLC system (AB SCIEX, Framingham, MA, USA). The freeze-dried samples were dissolved in deionized water, ionized, and detected through time-of-flight mass spectrometers (TOFMS) scans and product ion scans. UPLC analysis was performed through an ACQUITY UPLC BEH Shield RP18 (1.7 µm, 2.1 mm × 100 mm, Waters Corp., Milford, MA, USA). The mobile phase consisted of Solution A (0.1% *v*/*v* formic acid in water) and Solution B (0.1% *v*/*v* formic acid in acetonitrile). The separation step was carried out as follows: 2 min 95% *v*/*v* A, 15 min 65% *v*/*v* A, 18 min 20% *v*/*v* A, 23 min 20% *v*/*v* A, 24 min 95% *v*/*v* A, and 30 min 95% *v*/*v* A.

### 4.7. Lunasin Quantification by ELISA

The quantitative detection of lunasin was conducted according to a method reported previously [26]. A lunasin standard was dissolved in carbonic acid buffer (pH 9.6) and diluted with the range of 2.5–80 mg/L. LETW and PEWW were diluted in pure water. All samples were plated in 96-well plates and incubated for 2 h. Afterwards, they were blocked with 5% *w*/*v* nonfat dry milk TBST buffer at 37 °C for 1 h. Then, the plate was washed with TBST and 100 µL of lunasin rabbit polyclonal antibody (1:1000 dilution) was added to each well and incubated for 1 h. Immediately after, the wells were washed with TBST 3 times and the goat anti-rabbit IgG-HRP (1:5000) was added and incubated for 1 h. When the incubation finished, the plate was washed 3 times and was incubated with 100 mL of chemiluminescence solution at room temperature. The reaction was stopped with 100 µL 2 M H_2_SO_4_ for 30 min. The plate was read in a SpectraMax^®^ Plus 384 (Molecular Devices, San Jose, CA, USA) at 405 nm.

### 4.8. Cell Proliferation Assay

The human CRC cell line HT-29 was obtained from American Type Cell Collection (ATCC, Manassas, VA, USA) and maintained in RPMI1640 medium supplemented with 10% *v*/*v* FBS, 1% *v*/*v* penicillin, and 1% *v*/*v* streptomycin. Cells were grown in an incubator containing 5% *v*/*v* CO_2_ and 95% *v*/*v* air at 37 °C; the medium was changed every 24 h. All cells were assayed within 5–15 passages. Utilizing the CCK-8 kit (Dojindo, Kumamoto, Japan), the toxicity test was first performed. HT-29 cells (100 µL) were incubated in a 96-well plate (2.5 × 10^4^ cells/well) for 24 h, then the original medium was replaced with fresh medium containing different concentrations of samples. After 24 h of co-cultivation, the medium was discarded and washed with PBS twice, and a fresh medium with 10% *v*/*v* CCK-8 reagent was added to each cell. One hour later the absorbance (OD value) was determined by an enzyme-labeling instrument at a wavelength of 450 nm.

For the proliferation experiments, HT-29 cells were seeded into 96-well plates at a density of 1 × 10^4^ per well for 24 h and incubation was continued for 72 h. After that, the subsequent washing and CCK-8 reaction process were the same as that of the toxicity test. Finally, the absorbance was detected at 450 nm.

### 4.9. Real-Time Quantitative PCR (RT-qPCR)

After 24 h of co-cultivation with the medium containing 4 g/L LETW, total RNA extraction and cDNA synthesis of LETW treated HT-29 cells were performed as described in Section 4.2. mRNA expression was quantified by RT-qPCR as described in Section 4.4. Gene expression was normalized to the geometric mean of reference genes (GAPDH) using the 2^−^^ΔΔCt^ method.

### 4.10. Statistical Analysis

All data are expressed as the means ± standard deviation (SD). Differences among groups were determined by one-way ANOVA analysis and Duncan’s multiple range tests with the SPSS software (IBM, New York, NY, USA). Significant differences were expressed at *p* < 0.05 and *p* < 0.01.

## 5. Conclusions

In our study, the optimized *Agrobacterium*-mediated transformation of wheat provided a solution to compensate for the absence of lunasin in wheat. Lunasin was successfully expressed in wheat and the LETW exhibited enhanced anti-proliferative activity. Overall, we found that the lunasin extract from wheat was able to exert a similar bioactivity to the lunasin derived from soybeans. Trans-lunasin wheat has the potential as a functional food for cancer patients.

## Figures and Tables

**Table 1:** Primers used in this study.

Primer Name	Gene	Sequence (5′—3′)
Lunasin—F	Lunasin	ATGTCCAAGTGGCAGCACCAG
Lunasin—R	Lunasin	TCAGTCATCGTCATCGTCGTCAT
Lunasin—QF	Lunasin	AGCACCAGCAGCAGGATT
Lunasin—QR	Lunasin	GCCCTGAATCTTCTCCATGA
p21—F	p21	ATGAAATTCACCCCCTTTCC
p21—R	p21	AGGTGAGGGGACTCCAAAGT
Bax—F	Bax	CAAACTGGTGCTCAAGGCCC
Bax—R	Bax	CCGGAGGAAGTCCAATGTCC
Bcl-2—F	Bcl-2	TGGGATTCCTGCGGATTGAC
Bcl-2—R	Bcl-2	GTCTACTTCCTCTGTGATGTTGT
caspase-3—F	caspase-3	AGCGAATCAATGGACTCTGG
caspase-3—R	caspase-3	CCGAGATGTCATTCCAGTGC

## Supplementary Materials

The following are available online at https://www.mdpi.com/1422-0067/21/24/9594/s1, Figure S1. UPLC-MS/MS analysis of wild-type wheat. (a) The chromatogram of wild-type wheat. (b) Mass spectrum acquired from the peak at 10.510min in the chromatogram of wild-type wheat.
