# Supplementary material for: Overexpression of Soybean-Derived Lunasin in Wheat and Assessment of Its Anti-Proliferative Activity in Colorectal Cancer HT-29 Cells
Source: Int J Mol Sci. 2020 Dec 16;21(24):9594. doi: 10.3390/ijms21249594 (PMC7767187; doi:10.3390/ijms21249594)
Supplement: Supplementary file 1 [file ijms-21-09594-s001.pdf]

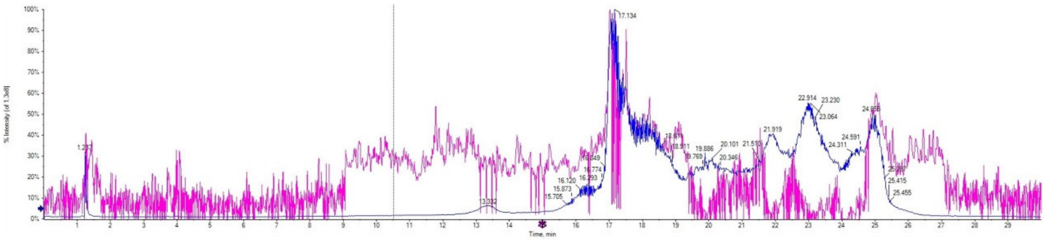

(a)

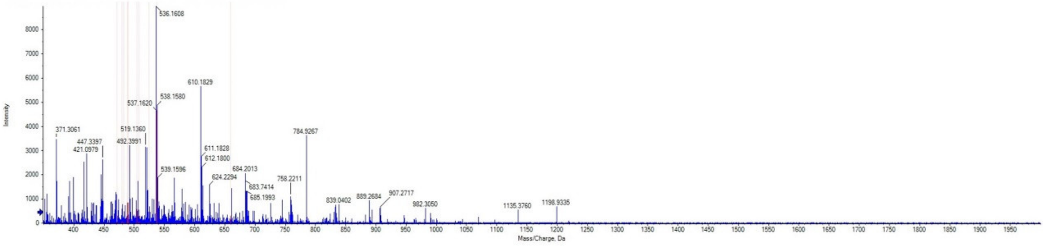

(b)

Figure S1. UPLC-MS/MS analysis of wild-type wheat. (a) The chromatogram of wild-type wheat. (b) Mass spectrum acquired from the peak at 10.510min in the chromatogram of wild-type wheat.
